# Differential Effects of RNA-Dependent RNA Polymerase 6 (RDR6) Silencing on New and Old World Begomoviruses in *Nicotiana benthamiana*

**DOI:** 10.3390/v15040919

**Published:** 2023-04-05

**Authors:** Emanuela Noris, Mattia Pegoraro, Sandra Palzhoff, Catalina Urrejola, Nicolai Wochner, Sigi Kober, Kerstin Ruoff, Slavica Matić, Vera Schnepf, Nina Weisshaar, Christina Wege

**Affiliations:** 1 Institute for Sustainable Plant Protection, National Research Council of Italy, Strada delle Cacce 73, 10135 Torino, Italy; 2 Institute of Biomaterials and Biomolecular Systems, Molecular and Synthetic Plant Virology, University of Stuttgart, Pfaffenwaldring 57, D-70569 Stuttgart, Germany

**Keywords:** geminivirus, RDR6, siRNAs, TYLCSV, AbMV, plant–virus interaction, RNA silencing, *Nicotiana benthamiana*, tissue tropism

## Abstract

RNA-dependent RNA polymerases (RDRs) are key players in the antiviral defence mediated by RNA silencing in plants. RDR6 is one of the major components of the process, regulating the infection of certain RNA viruses. To better clarify its function against DNA viruses, we analyzed the effect of RDR6 inactivation (RDR6i) in *N. benthamiana* plants on two phloem-limited begomoviruses, the bipartite Abutilon mosaic virus (AbMV) and the monopartite tomato yellow leaf curl Sardinia virus (TYLCSV). We observed exacerbated symptoms and DNA accumulation for the New World virus AbMV in RDR6i plants, varying with the plant growth temperature (ranging from 16 °C to 33 °C). However, for the TYLCSV of Old World origin, RDR6 depletion only affected symptom expression at elevated temperatures and to a minor extent; it did not affect the viral titre. The accumulation of viral siRNA differed between the two begomoviruses, being increased in RDR6i plants infected by AbMV but decreased in those infected by TYLCSV compared to wild-type plants. In situ hybridization revealed a 6.5-fold increase in the number of AbMV-infected nuclei in RDR6i plants but without egress from the phloem tissues. These results support the concept that begomoviruses adopt different strategies to counteract plant defences and that TYLCSV evades the functions exerted by RDR6 in this host.

## 1. Introduction

Geminiviruses cause extremely damaging diseases in different crops, including beans, tomatoes, maize, cotton, and cassava, especially in tropical and subtropical regions [1], resulting in up to 100% yield losses. These viruses are characterized by small, circular, single-stranded DNA (ssDNA) genomes of about 2.5–2.8 kb, encapsidated in twinned icosahedral particles. Based on genome organization, insect vectors, and host species, geminiviruses are now classified into fourteen genera [2], the largest of which is the genus *Begomovirus*, which is comprised of around 450 species having either monopartite or bipartite genomes [3]. Deviations in the arrangement and functions of their open reading frames (ORFs) have evolved between New and Old World begomoviruses depending on their geographical origin [4].

RNA silencing is a major defence mechanism exploited by higher organisms against invading nucleic acids, including viruses. It operates in a sequence-specific manner, targeting double-stranded RNA (dsRNA) molecules that are processed by DICER-like RNase III enzymes (DCLs) to produce small interfering RNAs (siRNAs) of 21–24 nucleotides (nt). siRNAs are incorporated into an RNA-induced silencing complex (RISC) made of several argonaute proteins (AGO), leading to specific recognition of complementary mRNAs [5]. Complementary mRNAs targeted by RISC are ultimately degraded; alternatively, siRNAs can be incorporated into the RNA-induced transcriptional gene silencing complex that directs chromatin modification and DNA methylation [6] through an RNA-dependent DNA methylation (RdDM) mechanism, resulting in transcriptional inhibition [7].

During infection by plant RNA viruses, dsRNA molecules originate from viral replication or transcription, while in the case of geminiviruses lacking dsRNA replication intermediates, dsRNAs are thought to arise from overlapping bidirectional transcripts or from hairpin-structured transcripts [8], eventually generating viral siRNAs (vsiRNAs) of 21–24 nt [9,10,11,12,13,14].

RNA silencing is amplified by the priming action of primary siRNAs on homologous RNA templates through the intervention of RNA-dependent RNA polymerases (RDRs), ultimately producing secondary siRNAs [15,16]. In plants, RDRs are involved in virus-induced, post-transcriptional gene silencing [5,17]. Six RDRs (RDR1 to RDR6) have been identified in *Arabidopsis* [18] that are engaged in developmental processes and stress-response and pathogen defence activities. From a functional point of view, no specific RNA silencing activities have been demonstrated for the RDR3–5 proteins, while RDR2 participates in the DCL3 interaction during transcriptional gene silencing (TGS) to produce 24 nt siRNAs [19] and is involved in the RdDM process [20,21]. RDR1 and RDR6 are considered major contributors to the post-transcriptional antiviral system [22]. Although RDR1 plays an important role in the antiviral defence of different host plants, including *Arabidopsis*, tomatoes, tobacco, and potatoes [23,24,25,26], it is nonfunctional in *Nicotiana benthamiana* due to the presence of a 72 nt insert that generates in-frame stop codons [27,28]. Such impaired RDR1 activity offers unique opportunities to investigate the specific antiviral roles of RDR6 in this host. In addition, RDR6 is involved in the production of trans-acting and natural antisense siRNAs [21].

Different groups have demonstrated the significance of RDR6 in the defence against RNA viruses [16,29] and viroids [30,31,32]. Specifically, it was shown that the depletion of RDR6 led to increased infection and accumulation of the cucumber mosaic virus (CMV) in *Arabidopsis* [29]. Similarly, RDR6-deficient *N. benthamiana* mutants over-accumulated different RNA viruses, such as the tomato chlorosis virus, potato virus X (PVX), and the tobacco mosaic virus, leading to reduced vsiRNA titres [16,29,33,34]. Interestingly, secondary siRNAs generated by RDR6 did not only boost systemic silencing signals but also permitted changes in the tissue localization of PVX [16] and the potato spindle tuber viroid (PSTVd) [31,32].

So far, the interaction of RDR6 with DNA viruses has been investigated in only few studies, with no systematic comparisons between mono- and bipartite Old and New World begomoviruses. In particular, a functional RDR6 was required for the induction of efficient virus-induced gene silencing in *Arabidopsis* when using a bipartite geminivirus-based vector [35]. More recently, it was shown that silencing of RDR6 led to the over-accumulation of three different monopartite begomoviruses in *N. benthamiana* [36]. Meanwhile, the interaction between geminiviral proteins and RDR6 protein or other components involved in the production of secondary siRNAs was also demonstrated, as is the case of the *AC2* protein of the bipartite mungbean yellow mosaic Indian virus, which inhibited RDR6 activity [37].

In this study, we sought to monitor, in parallel experiments, the role of RDR6 in the infection of mono- and bipartite begomoviruses—analyzing symptoms, viral DNA accumulation, and tissue tropism—in transgenic *N. benthamiana* plants having a constitutively silenced *NbRDR6* gene (RDR6i) [16]. As the temperature can influence the efficacy of silencing against foreign nucleic acids and viruses [33,38], we tested how temperature could alter the plant response to both viruses, in the context of RDR6 depletion. For these studies, the monopartite tomato yellow leaf curl Sardinia virus (TYLCSV) and the bipartite Abutilon mosaic virus (AbMV) were used, taken as representatives of Old and New World begomovirus species, respectively. TYLCSV, one of the agents causing the devastating tomato yellow leaf curl disease, affects tomato production in the Mediterranean basin [39] and constantly produces invasive recombinants characterized by increased pathogenicity [40,41,42,43,44]. AbMV, first found in *Abutilon sellovianum* var. *marmorata* (reviewed in [45]), can infect further ornamental plants, such as *Hibiscus spp.* and other *Abutilon* varieties and species [46,47], causing typical mosaic patterns with almost no growth reduction. AbMV may be introduced efficiently into further hosts, some of which develop severe symptoms [48]. Irrespective of their symptomatology, both AbMV and TYLCSV are strictly phloem-limited [49,50], possibly due to the intervention of tissue-specific promoters and transport proteins or the inefficient activation of the host’s replication machinery in non-phloem tissue [51,52]. For AbMV, however, the first evidence of its confinement to low numbers of cells through the plants’ silencing machinery came from experimental mixed infections with CMV and with transgenically provided CMV and AbMV gene products [49,53]. Subsequent comparative studies with AbMV and TYLCSV revealed that these two viruses responded differentially to co-infection with the tombusvirus artichoke mottle crinkle virus (AMCV) or the potyvirus cowpea aphid-borne mosaic virus (CABMV) and showed distinct reactions when inoculated in plants expressing the potent silencing suppressors produced by these RNA viruses, i.e., ACMV p19 and CABMV HC-Pro [50]. Such outcomes suggested that AbMV and TYLCSV could exploit different strategies to counteract plant defences. Consequently, the reactivity of both begomoviruses towards RDR6 inactivation was therefore studied under the same experimental conditions.

## 2. Materials and Methods

### 2.1. Plant Materials, Growth Conditions, and Virus Inoculation

*Nicotiana benthamiana* wt and RDR6i plants [16] were grown in soil in containment greenhouses with supplemental lighting at 24–26 °C during a 16 h light period (day) and at 20 °C during an 8 h dark period (night). For temperature-controlled studies, plants were maintained in growth chambers at constant temperatures of 16 °C, 23 °C, and 33 °C, with a photoperiod of 12 h of light and dark.

Plants were agroinoculated at the 4-leaf stage by injecting a suspension of *Agrobacterium tumefaciens* LBA4404 cells carrying the DNA-A and -B clones of AbMV (AbA and AbB, GenBank Acc. Nos. X15983/X15984) [54] or the TYLCSV clone (X61153 [55]). Control plants received bacteria carrying an empty pBIN19 plasmid or the AbB clone, respectively.

### 2.2. Symptom Analysis

Plants were scored for symptoms between 14 and 49 dpi at appropriate intervals, monitoring 4 to 10 plants per infection type and plant line, at each time point. Plant height was measured from the soil surface to the top of the apical bud. Lamina lengths were determined for the second and third subapical leaves collected from either group of test plants after 49 dpi (measuring 12 to 20 leaves per plant group).

### 2.3. Total Nucleic Acid (TNA) Extraction and Southern Blot Analysis

**Figure 1:**
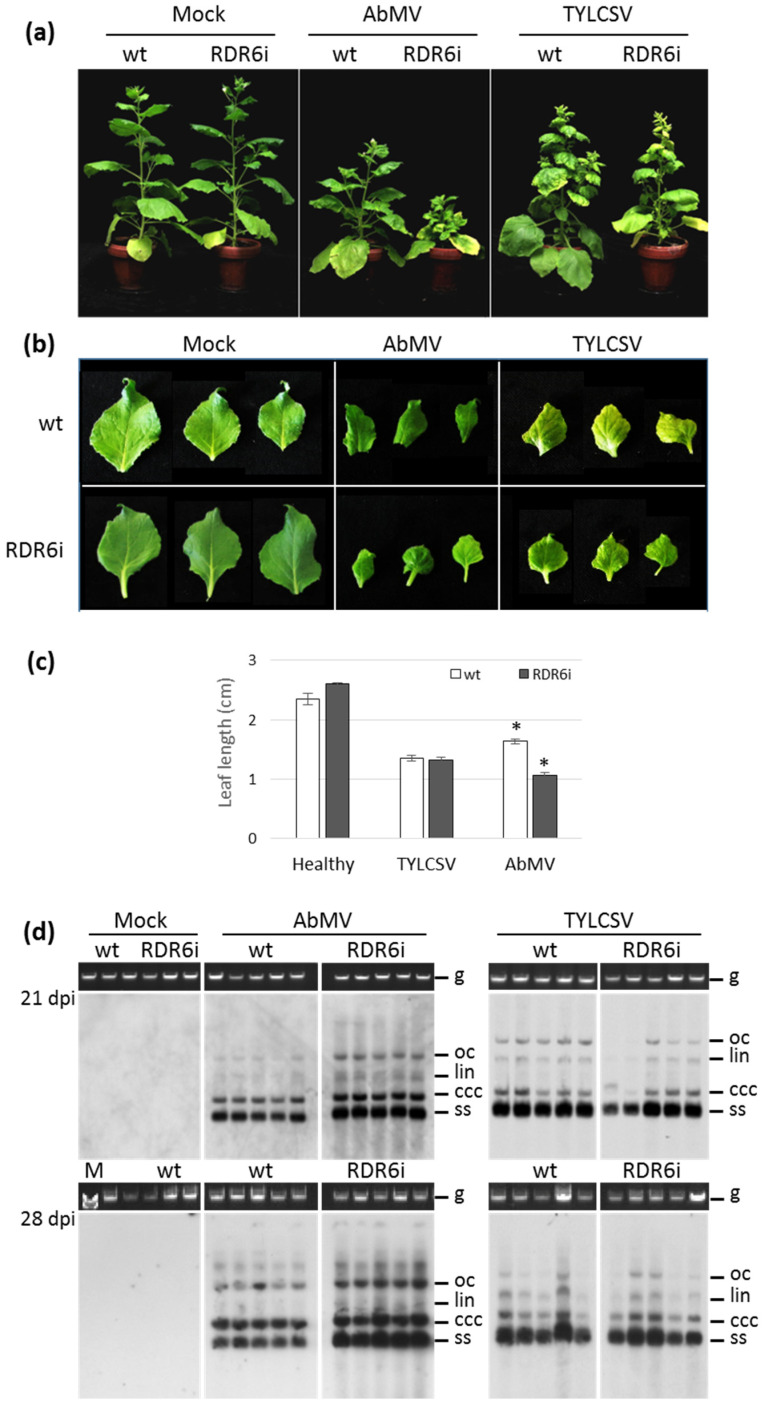
Impact of RDR6 depletion on AbMV or TYLCSV infection. (**a**) Comparison of symptom expression on wild-type (wt) and RDR6i *Nicotiana benthamiana* plants following mock inoculation or inoculation with either AbMV or TYLCSV. Photographs were taken 28 days post-inoculation (dpi). The inoculation assay was repeated eight times using more than 300 plants in total. (**b**) Second and third subapical leaves from mock-inoculated or AbMV/TYLCSV-infected plants, photographed at 49 dpi. Leaves are representative examples of 12–20 samples per infection type. (**c**) Length of leaves of mock-inoculated or AbMV/TYLCSV-infected plants, measured at 49 dpi. Measurements were conducted on 12–20 leaves per treatment for each genotype. Asterisks indicate significant differences among treatments according to the Student’s *t*-test (*p* < 0.01). (**d**) AbMV and TYLCSV genomic DNA accumulation in wt and RDR6i plants, evaluated by Southern blot on total DNA extracted from plants at 21 or 28 dpi, as indicated. gDNA, total genomic DNA, is shown as the loading control; oc, lin, ccc, and ss represent the open circular, linear, covalently closed circular supercoiled dsDNA, and single-stranded circular ssDNA forms, respectively. M: DNA molecular weight marker.

**Figure 2:**
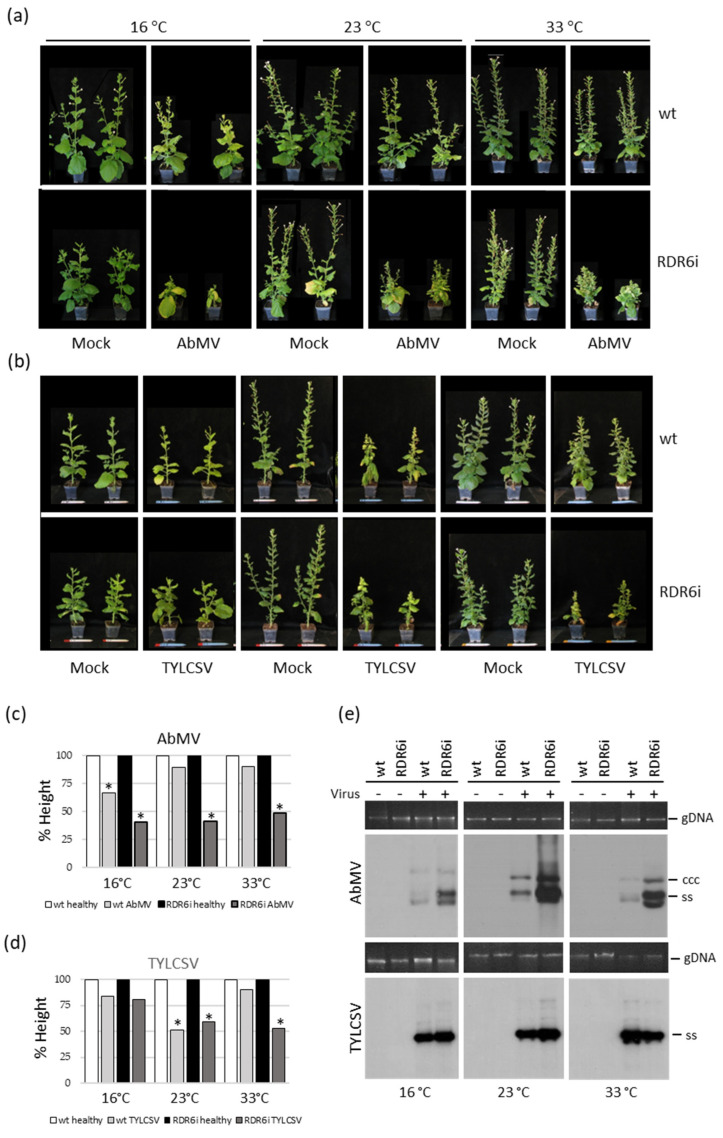
Impact of plant cultivation temperature and RDR6 depletion genotype (RDR6i) on AbMV and TYLCSV infection. (**a**,**b**) Symptom expression on wild-type (wt) and RDR6i *Nicotiana benthamiana* plants infected by either (**a**) AbMV or (**b**) TYLCSV, grown at constant temperatures of 16, 23, and 33 °C. Mock-inoculated plants served as controls. Photographs were taken at 49 dpi. Inoculation assays were repeated twice, using a total of 10 plants per genotype and infection type. (**c**,**d**) The height of wt and RDR6i plants mock-inoculated or infected with either (**c**) AbMV or (**d**) TYLCSV, measured at 49 dpi. The height of mock-inoculated plants (10 plants per treatment) was set to 100%. Asterisks denote statistically significant differences according to Student’s *t*-test (*p* < 0.01), comparing infected vs. mock-inoculated plants for each genotype and growth condition. (**e**) AbMV and TYLCSV genomic DNA accumulation in wt and RDR6i plants was evaluated using a Southern blot on total nucleic acids (TNAs) extracted from individual representative plants at 49 dpi. The symbols “+” and “−“ indicate the presence or absence of the virus, respectively. gDNA, total genomic DNA, is shown as the loading control; ccc and ss represent covalently closed circular supercoiled dsDNA and single-stranded circular ssDNA forms, respectively.

TNAs were extracted from immature leaves (10–20 mm in length) using a phenol-chloroform-based method [49] and quantified using a NanoDrop ND-100 spectrophotometer (Peqlab GmbH, Erlangen, Germany). Virus accumulation was evaluated through Southern blot analyses after separating 500 ng of TNAs per lane in 1% agarose gels in TBE, either in the presence (Figure 1) or absence (Figure 2) of 0.5 μg/mL ethidium bromide. TNAs transferred onto nylon membranes were hybridized with DNA probes specific for either the full-length AbMV DNA-A or the TYLCSV CP gene sequence, respectively, as described by [49,50]. Probes were labelled with digoxigenin via a DIG-High Prime kit and detected through CSPD or CDP Star chemiluminescence, following the manufacturer’s instructions (Roche Deutschland Holding GmbH, Penzberg, Germany). Signals were visualized on X-ray films (Fujifilm, Reutlingen, Germany) and documented using a transmitted light scanner.

### 2.4. In Situ Hybridization (ISH)

To examine viral tissue tropism at high resolution, virus DNA was detected in ribbons of typically 8–12 serial 7.5 µm sections of formaldehyde-fixed, Paraplast Plus^®^ (Merck KGaA, Darmstadt, Germany)-embedded explants via the ISH of biotinylated DNA probes and stain precipitates, as described in detail in our earlier work [50]. For visualizing virus distribution patterns in organs and determining the numbers of infected nuclei, fluorescent ISH (FISH) was performed according to [56]. Apical bud explants or 100 µm thick stem cross-sections cut by a hand microtome (model 503000; Dr. G. Schuchardt, Göttingen, Germany) were prepared from fresh material collected at 20–30 days post-inoculation (dpi), fixed for 1–2 h, and washed as described by [56]. The hybridization was conducted using 5′-terminally Cy3-labelled oligonucleotide probes with comparable T_M_ values (Biomers, Ulm and Biometra, Göttingen, Germany) diluted in hybridization buffer (0.01 pmol/μL). The primers used were: for AbMV ‘NC_001928-AbA-reverse_AC1’, 5′-GGT TTC CCC TCT CCT CTT TC-3′; for TYLCSV ‘TY-It_V1’, 5′-ACT GGG TTA GAA GCA TGA GTA C-3′. A negative control primer was directed against a eubacterial marker sequence ‘C20’: 5′-GGT AAG GTT CTG CGC GTT-3′. Following hybridization for 12–16 h at 25 °C in a humid chamber, specimens were evaluated and documented using a Zeiss Axiophot microscope (Carl Zeiss, Jena, Germany), with filter combination AHF F36-503 HC for Cy3/TRITC (AHF Analysentechnik, Tübingen, Germany) for fluorescence detection, and a Canon Powershot G1 digital camera (Canon Europa N.V., Amstelveen, The Netherlands) adapted to the microscope.

### 2.5. Viral siRNA Detection by Northern Blot Analysis

SiRNAs were isolated from plant tissues using a NucloSpin miRNA isolation kit (no. 740971.50; Macherey-Nagel, Düren, Germany), using aliquots of 50 mg leaf material homogenized in liquid nitrogen, according to the manufacturer’s protocol. Small RNAs (<200 nt) were enriched through the recommended buffer conditions and eluted in 30 µL RNase-free H_2_O. Depending on the probe sensitivity, 0.1–1 µg total small RNA per lane (as determined by UV spectroscopy, with equal amounts of RNA per virus within each experiment) were used for Northern blot analysis, essentially according to [57]. Samples were dried at 60 °C and resuspended in 8 µL of formamide RNA loading reagent, heated to 95 °C for 3 min, and separated in 7 M of urea-containing 18% polyacrylamide gels in TBE. MicroRNA size markers (NEB N2102S; New England Biolabs, Ipswich, MA, USA) and hybridization specificity DNA oligonucleotide standards (‘NC_001928-AbA-Pos1977C’: 5′-GTG ATT CAA GGA CAG GGA AGA C-3′ or ‘X61153-TYLCSV-Sar-Pos1952C’: 5′-CAG CCG GAC AGG AAA GAC AAC-3′) were used for control purposes. Gels were stained with Sybr^®^Gold (Invitrogen, Paisley, UK), documented in a ChemiDoc XRS system (Bio-Rad, Munich, Germany), and washed in RNase-free H_2_O. The lower third of the gel (containing siRNAs) was subjected to semi-dry electro-blotting onto Hybond-NX nylon membranes (Amersham/GE Healthcare, Amersham, UK). RNAs were then linked to the membranes by 1-ethyl-3-(3-dimethylaminopropyl) carbodiimide (EDC) [58] prior to hybridization with digoxigenin-labelled DNA probes for Southern analysis (see Section 3.3). Hybridization at 40 °C overnight was conducted according to [59], with minor modifications (personal communication Chikara Masuta, University of Hokkaido, Sapporo, Japan). These consisted of the addition of 15 mM of Na_2_HPO_4_ (adjusted to pH 7.0 with H_3_PO_4_) to both the prehybridization and hybridization mixture [59], the use of random primed, labelled probes (obtained through a DIG-High Prime kit, as indicated above, denatured in the hybridization mixture prior to use at 80 °C for 10 min), and over-night hybridization. Post-hybridization washes used 2 × SSC, 0.1% SDS; signals were visualized via CSPD, as described above for Southern blots.

### 2.6. Statistical Analysis

Statistical analyses were conducted with either the program “R” (The R Foundation for Statistical Computing, Version: 2.12.0), or SigmaStat software (for Windows, version 1.0, 1992–94 Jandel corporation, 1993 MicroHelp, Inc., Fall Creek, WI, USA, and Heiler Software GmbH, Stuttgart, Germany), or Microsoft Excel software (Windows 11 Pro). All values were tested first for normal distributions using the Shapiro test to calculate the mean values for each infection type within each plant genotype. Significant differences between the test groups were validated using Student’s *t*-test and analyses of variance based on one-way and/or two-way ANOVAs with post-hoc Tukey tests for significance.

## 3. Results and Discussion

### 3.1. Silencing of NbRDR6 Has a Differential Effect on the Symptoms Induced by AbMV and TYLCSV and on Their Genome Accumulation

To evaluate the role of *NbRDR6* in infections with AbMV or TYLCSV, a series of experiments was carried out in greenhouses, where only moderate control of light and temperature regimes could be accomplished. Under these conditions, the size and growth of *N. benthamiana* RDR6i plants were similar to wild-type (wt) plants, as reported [16]. Following AbMV and TYLCSV agroinoculation, all plants of both wt and RDR6i genotypes became infected. As more than 300 plants, with four to ten plants per virus and plant line in eight independent experiments in two greenhouses, were examined, we concluded that RDR6 was not directly involved in preventing systemic infection by these viruses, at least in this host.

The characteristic dark-green/light-green mosaic pattern and leaf rolling induced by AbMV on *N. benthamiana* were manifest on all inoculated wt plants, starting about 14 dpi, and persisted until the end of the experiments (42 or 54 dpi) (Figure 1a). At this time, the RDR6i plants infected by AbMV appeared considerably more stunted than their wt counterparts (Figure 1a). During the experiments, the growth of RDR6i plants infected with AbMV was increasingly retarded compared to wt controls, reaching up to 50–75% height reduction at 42 days (Figure 1a; Table 1). Moreover, a quantitative evaluation of leaf size revealed that the second and third subapical leaves of AbMV-infected RDR6i plants were about 35% shorter than those of wt individuals (Figure 1b,c).

In the case of TYLCSV infection, a slight inhibition of plant growth occurred from 14 dpi onward for both wt and RDR6i genotypes, with initial onset of leaf yellowing and curling. However, up to 54 dpi, no evident symptom variations were noticed between the two genotypes, and neither leaf size nor plant height developed statistically significant differences (Figure 1a–c, Table 1, and data not shown).

To investigate whether there were changes in symptom severity or, generally, to elucidate how the depletion of the *NbRDR6* gene product activity could impact the accumulation of viral DNA, total nucleic acids (TNAs) extracted from individual plants were analysed using Southern blots with virus-specific probes. Increased amounts of AbMV genomic and replicative DNA forms were consistently obvious for RDR6i plants compared to wt plants, while no differences in TYLCSV DNA accumulation were recorded between the two genotypes (Figure 1d). The blot analyses with an indirect enzyme-based system and luminescence detection via film exposure, however, did not allow for reliable quantification of the AbMV DNA titre enhancement. Hence, we decided to determine the number of virus-accumulating nuclei in all virus–plant genotype combinations, as described in Section 3.3 below.

The strong exacerbation of AbMV symptoms and the increase in virus titre are in agreement with previous reports of *Arabidopsis* plants compromised in *RDR6* gene expression that were hyper-susceptible to cabbage leaf curl virus, a bipartite begomovirus of New World origin, and supported a 10–20% increase in viral accumulation [35]. More severe symptoms and up to a 3-fold-increased viral load were also described for *RDR6*-silenced *N. benthamiana* plants challenged with three different monopartite Old World begomoviruses, i.e., the tomato yellow leaf curl China virus, the tobacco curly shoot virus, and the tomato leaf curl Yunnan virus [36], thus supporting the defensive role of RDR6 in this host.

In the case of TYLCSV, the results obtained here (no symptom exacerbation and no increase in the viral titer of RDR6i plants) require a re-evaluation of the role of RDR6 in the defence against this virus, as they are in contrast with those reported for three other monopartite viruses [36], thus indicating that RDR6 inactivation did not provide additional support for TYLCSV infection. Nonetheless, since neither hyper-susceptibility nor overaccumulation of TYLCSV occurred in this RDR6i line, a residual RDR6 activity sufficient to mount a counteracting effect towards this monopartite begomovirus cannot be excluded. Further experiments with the *N. benthamiana rdr6* mutant lines recently obtained by the CRISPR/Cas9 genome editing system [60,61] would help to clarify this aspect.

### 3.2. Plant Growth Temperature Influences the Response of RDR6i Plants to AbMV but Not to TYLCSV Infection

Since temperature can influence the silencing-mediated defensive response of plants against transgenes and viruses [33,38], we investigated whether and to what extent plant growth temperature impacted the infections of the two begomoviruses in the context of RDR6 depletion. A series of inoculation experiments was conducted under controlled and constant temperatures, at 16 °C, 23 °C, and 33 °C, representing conditions where silencing activity is expected to be reduced or increased. In these experiments, except for the reduced growth of mock-inoculated RDR6i plants at 16 °C compared to wt individuals, no evident differences manifest in the size or growth habits of RDR6i plants at the other temperatures (Figure 2a,b).

Regarding the results of virus inoculation, in the case of AbMV, considerable symptom exacerbation occurred in wt plants grown at 16 °C, starting at 14 dpi (not shown), compared to the other growth temperatures; this effect became more intense with time progression (up to 49 dpi) (Figure 2a). These observations matched up to 66% inhibition of plant growth occurring at 16 °C, but not at the other temperatures tested (Figure 2c). However, in the case of RDR6i plants, symptom exacerbation, accompanied by a reduction in plant growth of more than 50% was independent of the temperature (Figure 2a,c).

On the other hand, wt plants infected with TYLCSV appeared more stunted and about half the height of mock-inoculated controls only when grown at 23 °C (Figure 2b). Regarding RDR6i plants, enhanced growth inhibition compared to infected wt controls was noticed, ranging between 58 and 52% for plants maintained at 23 °C and 33 °C, respectively (Figure 2b,d).

Interestingly, a remarkable increase in the AbMV DNA titre was manifest in RDR6i plants compared to wt at all the temperatures tested (Figure 2e, upper panel). By contrast, despite slight variations among the individual plants of both genotypes infected by TYLCSV, no dramatic differences in viral DNA accumulation could be noticed between wt and RDR6i plants grown at any of the temperatures tested (Figure 2e, lower panel).

Regarding the response of AbMV in RDR6i plants, both symptom exacerbation and increased virus titres were manifest independently of the growth temperature and also at the lowest temperature tested, at which the antiviral RNA silencing is already intrinsically impaired [38]. Overall, the exacerbation of AbMV symptoms in RDR6i plants recalls previously reported results obtained with RNA viruses in plants with depleted RDR6 function, grown at elevated temperatures [33]. Conversely, TYLCSV infection appeared uncoupled from either temperature or RDR6i depletion. The symptom exacerbation observed at 23 °C and 33 °C, without significant changes in virus titres, points to an intervention of other molecular events in the interaction between TYLCSV and this plant host.

### 3.3. In Situ Hybridization (ISH) Reveals Increased Numbers of Infected Nuclei in RDR6i Plants with AbMV but Not TYLCSV

To scrutinize the origin of the AbMV DNA titre increase in RDR6i plants, we investigated through ISH whether the number and distribution of nuclei containing viral DNA were altered compared to wt specimens, or if the enhanced signals on blots resulted from higher replication within cells. Samples from the TYLCSV-infected plants of both genotypes, which showed similar virus accumulation levels, were also analysed in parallel. Due to the overall limited percentages of nuclei infected by these phloem-limited viruses in wt *N. benthamiana* (about 1% of leaf nuclei for AbMV [62] and up to about 7% for TYLCSV [41]), numerous consecutive sections of stems, leaves, apical, and flower buds had to be examined to ensure correct localization and statistically significant quantitative data. For the optimal identification of cell and tissue types, virus DNA was detected using precipitated stains in semi-thin sections, as described in [50]. In order to count statistically meaningful numbers of infected nuclei in larger explants and to visualize distribution patterns, FISHwas performed using the ten times thicker hand microtome sections of the stems or whole flower buds. Specimens from AbMV DNA-B mock-inoculated plants and infected ones treated with equally labelled, non-homologous probes served as negative controls and ensured the specificity of the signals.

**Figure 3:**
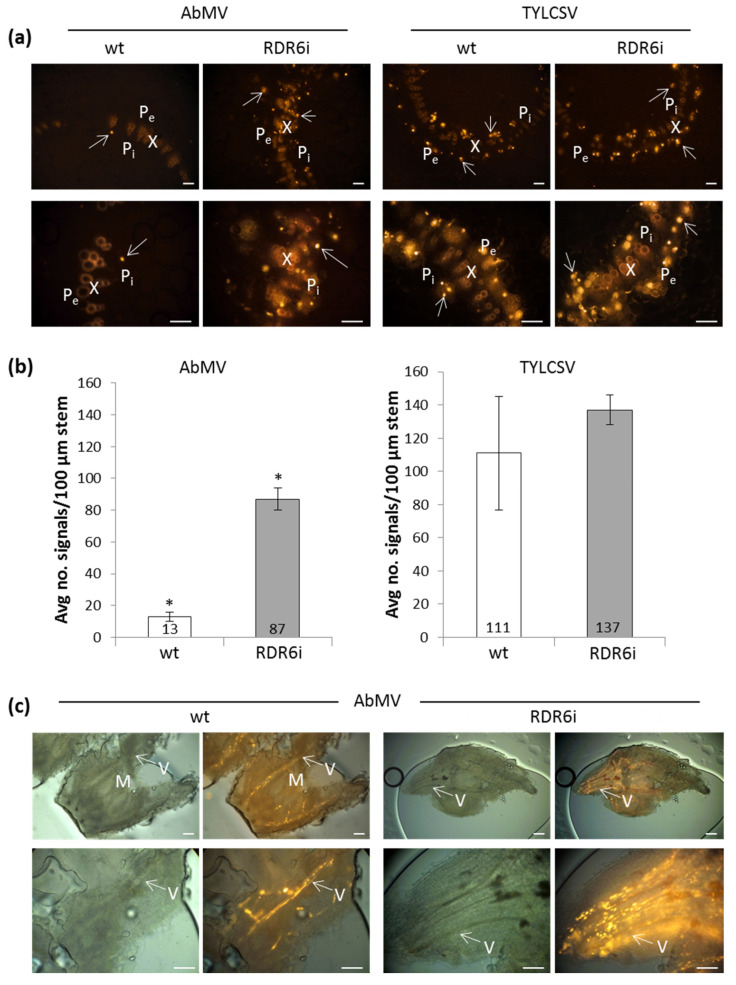
Localization of AbMV or TYLCSV DNA in leaves of wild-type (wt) and RDR6i plants. (**a**) FISH analysis of stem cross sections (100 µm thick) fixed at 49 dpi; virus detection using fluorescently labelled AbMV- or TYLCSV-specific oligonucleotide probes. Arrows point at signals representing exemplary virus-infected nuclei inside the two (internal and external) phloem domains adjacent to the auto-fluorescent xylem elements. X: xylem, P_i/e_: phloem (internal/external), scale bars: 100 µm. (**b**) Average numbers of virus-infected nuclei in 100 µm thick stem sections of similar diameters were measured in plants infected by either AbMV or TYLCSV. Data represent 673 signals for AbMV (six or eight sections per plant genotype), and 1154 signals for TYLCSV (three or six sections, respectively). Asterisks indicate significant differences, as indicated above. (**c**) AbMV DNA in vascular tissues around the apical meristem (for wt plants) or flower bud (for RDR6i plants) of whole-mount specimens. M: meristem, V: vascular bundle (exemplary labels), scale bars: 100 µm.

For both viruses and both plant genotypes, all signals in more than 250 sections per infection type were found to be associated with the vascular tissues. In wt plants, AbMV DNAs were exclusively detected in the phloem, as previously described by [49,50,63,64]. This virus also remained confined to this tissue in RDR6i plants (exemplary data, only from the FISH experiments, are shown in Figure 3). In line with the enhanced DNA accumulation recorded by Southern blot analyses, a 6.5-fold increase in the number of AbMV-infected nuclei of phloem parenchyma and companion cells was found in the stem cross-sections of RDR6i plants compared to their wt counterparts (Figure 3a,b). In contrast, no significant differences were observed between the numbers of TYLCSV-infected nuclei in RDR6i or wt plants, respectively (Figure 3a,b), in agreement with the Southern blot hybridization results.

FISH of the whole-mount specimens of the apical meristems and flower buds of AbMV-invaded wt and RDR6i plants are shown in Figure 3c. These analyses, however, did not allow for the quantification of viral DNA within single nuclei; therefore, our findings do not rule out an additional amplification of the AbMV genome in individual cells under RDR6i conditions.

These results confirm, once more, that AbMV and TYLCSV genomic DNAs are confined to the phloem [50]. Such tissue limitation was not overcome by the overexpression of strong silencing suppressor proteins of different unrelated RNA viruses in earlier studies, although it was partially released only for AbMV through co-infection with the respective viruses [49,50]. The latter demonstrated that, in principle, AbMV is able to cross the phloem boundary so that viral confinement to the vascular tissue is likely to result from antiviral silencing exerted in the mesophyll, although a role in complementing further RNA-viral gene products cannot be fully excluded. Here, despite the strong increase in the number of infected nuclei observed for AbMV in RDR6i plants, no phloem evasion occurred, indicating that (i) the symptom enhancement of AbMV was independent of mesophyll invasion and (ii) RDR6 seemed not to be the key component of the plants’ surveillance system underlying the tissue localization of these begomoviruses.

### 3.4. Depletion of NbRDR6 Gene Expression Modulates the Amount of Virus-Derived siRNAs

**Figure 4:**
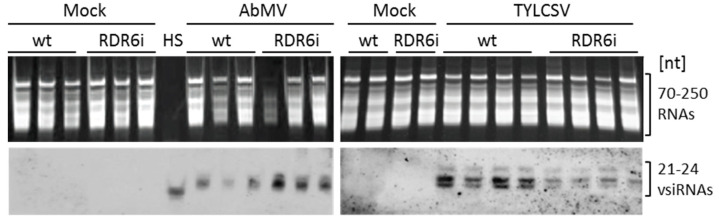
Northern blot analysis of virus-derived siRNAs in wild type (wt) or RDR6i *Nicotiana benthamiana* plants, either mock-inoculated (control) or infected by either AbMV or TYLCSV. vsiRNAs were detected using PCR-amplified, digoxigenin-labelled, full-length virus DNA probes via chemiluminescence. Small RNA (≈70 to ≈250 nt apparent lengths) loads of the corresponding denaturing polyacrylamide gels (**above panel**, Sybr^®^ Gold stain) are shown as loading control. HS, an AbMV-derived DNA oligonucleotide (from ORF C1), was used as a hybridisation specificity standard.

To obtain a better understanding of the role of RDR6 during AbMV/TYLCSV infection, the functionality of RDR6 was investigated by analysing the abundance of vsiRNA accumulated at the end of the infection experiments (49 dpi) in plants grown under greenhouse conditions. Compared to wt plants, moderately increased intensities of hybridization signals indicated elevated amounts of AbMV-derived siRNAs in RDR6i plants, while the opposite was observed for TYLCSV-derived siRNAs (Figure 4). The technically challenging vsiRNA separation and detection, however, did not allow for reliable quantification. Increased titres of AbMV-derived siRNA in RDR6i plants might result from an over-accumulation of the viral genome (Figure 1d) and, consequently, of viral mRNAs in an elevated number of cells (Figure 3). This would be similar to findings reported for PSTVd, where the compromised *rdr6* gene expression in *N. benthamiana* led to elevated amounts of viroid-derived siRNAs [31,65].

Conversely, reduced levels of TYLCSV siRNAs in RDR6i plants (Figure 4) might be explained by an absence of viral DNA overaccumulation upon the combined depletion of RDR6 and RDR1 activities. Nonetheless, it is interesting to note that such a decrease occurred regardless of the viral titres, which would confirm that TYLCSV infection is not influenced by RDR6. This would suggest that plants adopt other RDRs, other mechanisms involving superordinate regulatory systems, or different independent pathways for the defence against this monopartite virus.

## 4. Conclusions

The establishment of viral infections in plants results from the equilibrium of viral weapons, host defences, and viral counter-defences. The antiviral defence based on post-transcriptional gene silencing (PTGS), raised by dsRNA molecules, relies on the production of primary siRNAs that lead to the degradation of viral ssRNAs and the subsequent production of secondary siRNAs, thus amplifying the silencing process and rendering it transitive. RDR6 is the major contributor to the amplification step of RNA silencing. In this work, the role of RDR6 in the infection of the bipartite AbMV and the monopartite TYLCSV was examined using RDR6i *N. benthamiana* plants. The remarkable differences observed between the two begomoviruses suggest that they adopt different strategies to overcome the host antiviral silencing responses and, specifically, the amplification step of silencing. Overall, these results confirm our previous observations regarding the different reactions of AbMV and TYLCSV to co-infection with RNA viruses expressing silencing suppressors that bind and interfere with siRNA and miRNA activity [66,67] or their response when inoculated onto transgenic plants overexpressing silencing suppressors [50]. These data also indicate that PTGS affects, in a different way, the two begomoviruses, being inefficient in counteracting TYLCSV but active against AbMV. Aside from its role in siRNA amplification, RDR6 is also involved in non-canonical TGS pathways, linking DNA methylation and PTGS; in this regard, RDR6 participates in the initiation of cytosine methylation, taking part in an RdDM pathway against transposable elements [68,69,70]. Although the ability of RDR6-RdDM to reestablish epigenetic silencing and induce de novo DNA methylation on geminiviruses has yet to be demonstrated, our results suggest that this pathway might only function against AbMV, but not—primarily—against TYLCSV.

As a counteractive response to silencing-based plant defence, different geminiviral proteins have been found to have silencing suppressor properties, acting both at the PTGS and TGS levels in the formation, amplification, or effector steps of the process or in counteracting RdDM, respectively [71]. In particular, the replication-associated protein (Rep) of a TYLCSV isolate was found to repress genes involved in the maintenance of methylation, acting as a suppressor of TGS [72]. In addition, the V2 protein of the closely related species tomato yellow leaf curl virus interferes with an RDR6 co-factor, the Suppressor of Gene Silencing 3 (SGS3) [73] and interacts with the putative histone deacetylase HDA6 and with AGO4 [74,75]. However, no such activities have been investigated for the corresponding V2 protein of TYLCSV. Remarkably, no AV2 protein is encoded by the New World virus AbMV and no indication of silencing suppression activities of any AbMV protein has been available up to this point.

In the scenario described in this manuscript, we could speculate that another RDR may be the major player active in TYLCSV defence, that a pathway of overriding importance is additionally operating, or that alternative defence strategies are switched on against this virus, such as TGS. Assuming this latter hypothesis for situations where both RDR6 and RDR1 are missing or insufficiently active, it would be interesting to evaluate whether the TGS process mediated by RdDM triggered by RDR2 is functional against TYLCSV, as it is the case for pepper-infecting geminiviruses [76]. Nonetheless, clarifying the resources mounted by TYLCSV to evade the amplification step of PTGS or the RdDM-RDR2/6 pathways would be important to deepen our understanding of the relationships between TYLCSV and its hosts, and possibly to develop successful strategies aimed at reducing its impact in agriculture.

## Figures and Tables

**Table 1:** Effect of RDR6 depletion on the height of RDR6i *Nicotiana benthamiana* plants during infection with either AbMV or TYLCSV, compared to wild-type plants.

Virus Inoculum	Days Post-Inoculation (dpi)		
	19	31	42
AbMV	0%	0–25%	50–75%
TYLCSV	0%	0%	0%

Data represent the average reductions in the height (%) of virus-infected RDR6i plants vs. wild-type controls [i.e., the extent of stunting], measured at different times post-inoculation.

## Data Availability

All relevant data are available in the manuscript.
